# Epidermoid cyst of clitoris mimicking clitoromegaly

**DOI:** 10.4103/0971-9261.69137

**Published:** 2010

**Authors:** Satish Kumar Aggarwal, Vivek Manchanda, Nitin Pant

**Affiliations:** Department of Pediatric Surgery, Maulana Azad Medical College and Associated Lok Nayak and GB Pant Hospitals, New Delhi, India

**Keywords:** Clitoral cysts, clitoromegaly, epidermoid cysts

## Abstract

Clitoromegaly in pediatric and adolescent age group is usually indicative of a disorder of sexual differentiation. We report a girl child presenting with clitoral enlargement due to an epidermoid cyst. The cyst was excised with complete cosmetic recovery.

## INTRODUCTION

Clitoromegaly (or macroclitoris) due to nonendocrinal causes is rare and rarer still is clitoral enlargement due to some underlying mass lesion, such as a pilonidal cyst, epidermoid cyst, or an abscess. A good clinical examination can spare the child from extensive investigations and associated emotional disturbances in such cases.

## CASE REPORT

**Figure 1:**
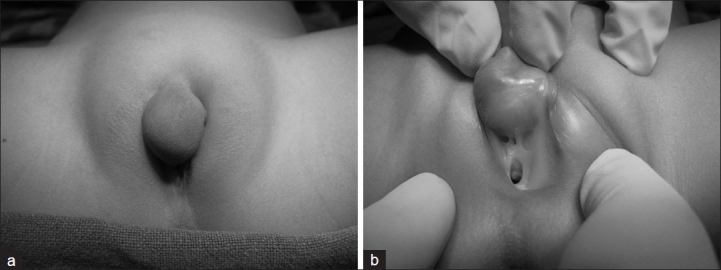
(a) Clinical photo showing clitoromegaly; and (b) extent of the cyst

**Figure 2:**
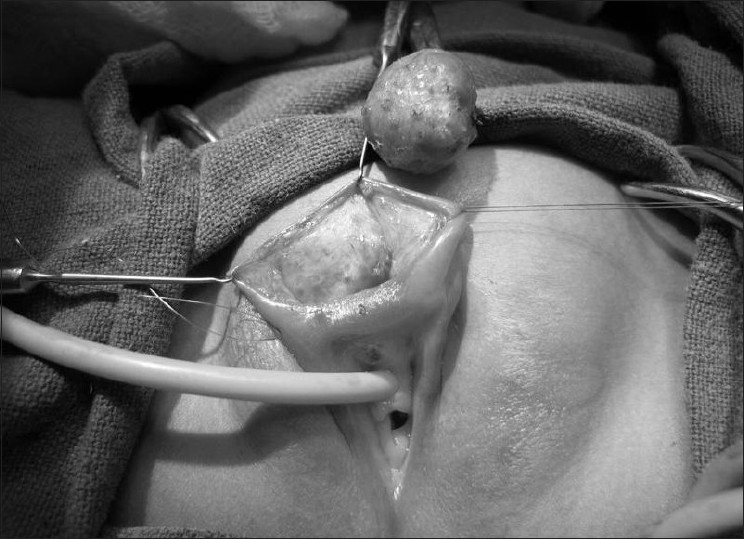
Surgical excision of the cyst

**Figure 3:**
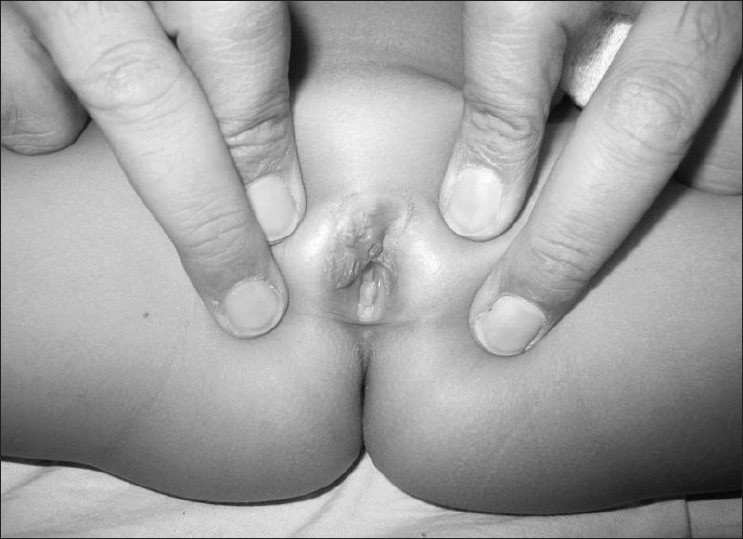
Postoperative appearance 4 months after surgery

A 5-year-old girl presented with progressive clitoral enlargement of 1 year. There was no urinary complaint, history of trauma, or hormonal treatment. There were no systemic complaints. General physical and abdominal examination was unremarkable. External genitalia were female type with clitoral enlargement. A cystic mass 4 × 3 cm was noted over the clitoris [Figure 1a and b].The labia minora on right were splayed over the cyst. Urethral and vaginal openings were separate. There was no hyperpigmentation. Ultrasound revealed normal urinary tract and internal genitalia. The cyst was excised under general anesthesia [Figure 2]. The histopathology revealed it to be a keratinous epidermoid cyst. The child recovered uneventfully. Normal cosmetic appearance of the external genitalia was restored [Figure 3]. There is no recurrence at 30-month follow-up.

## DISCUSSION

Female external genitalia may have multitude of cysts. Merlob *et al* gave an incidence of 0.6% in infancy.[1] Such cysts may be vaginal (hymenal), paraurethral, or clitoral. Clitoral cysts are most infrequent among these.

Clitoral cysts present clinically as swelling on clitoris. The literature is replete with instances when such a cyst has been diagnosed clinically as clitoromegaly with differential diagnosis of true hermaphroditism, adrenal hyperplasia, clitoral, ovarian, and adrenal neoplasms, stromal hyperthecosis, polycystic ovarian syndrome, and exogenous androgen exposure.[2] Such errors in diagnosis mandate many laboratory investigations, such as serum levels of free testosterone, dehydroepiandrosterone sulfate, 17-hydroxyprogesterone, total testosterone, androstenedione, deoxycorticosterone, 11-deoxycortisol, karyotype, an intravenous pyelogram, and pelvic ultrasound. This is time consuming and expensive. A simple clinical examination should be sufficient for differentiating clitoral cyst from the hormonal causes of clitoromegaly.[3]

Epidermoid cysts of the clitoris are seen commonly after type I female genital mutilation/female circumcision done in some ethnic communities in Africa and West Asia.[4,5] A few cases of such cysts in infancy have also been reported.[1,3,6] A simple enucleation of the cyst with reconstruction of external genitalia is the preferred mode of treatment.

A good clinical examination should be the first investigation in clitoromegaly. Clitorial enlargement due to hormonal causes or disorders of sexual differentiation is symmetrical and uniform, which it is not in this case. Clitoral cysts or other local lesions need to be considered, which may avoid unnecessary investigations.
